# Title: β3 Adrenergic Receptor Signaling in the Human Myometrium

**DOI:** 10.1007/s43032-022-00917-y

**Published:** 2022-04-04

**Authors:** Hazik Asif, Scott D. Barnett, Iain L. O. Buxton

**Affiliations:** School of Medicine, Department of Pharmacology, Myometrial Function Laboratory, University of Nevada, Reno, NV 89557-0318 USA

**Keywords:** β3 adrenergic receptor, Mirabegron, Preterm Labor, Myometrium, Nitric Oxide, Endothelial Cells

## Abstract

Preterm labor leading to preterm birth is the leading cause of infant morbidity and mortality. Although β2 adrenergic agonists fail to provide adequate tocolysis, the expression of the β3 adrenergic receptor in myometrium and its unique signaling suggest a role for β3 agonist in the management of preterm labor. Western blot analysis showed that the β3 adrenergic receptor expression increased in human pregnancy myometrium compared to nonpregnant tissues (p < 0.0001). There was no difference in β3 adrenergic receptor expression throughout pregnancy (p > 0.05). The addition of the β3 agonist mirabegron in the tissue bath relaxed oxytocin contracted myometrium with an EC_50_ of 41.5 µM. Relaxation was partially blocked by the addition of the eNOS blocker Nω-nitro-L-arginine, or the large conductance potassium channel blocker paxilline. Combination of Nω-nitro-L-arginine and paxilline prevented mirabegron-mediated relaxation. Imaging revealed that the β3 adrenergic receptors are expressed by both myocyte and microvascular endothelial cells isolated from human myometrium. Nitric oxide production measured by 4-amino-5-methylamino-2',7'-difluorofluorescein diacetate revealed that mirabegron stimulated nitric oxide production in myometrial endothelial cells. These data suggest that both endothelial and smooth muscle cells contribute to relaxation through disparate signaling pathways. Repurposing of approved medications tested in human myometrium as uterine tocolytics can advance prevention of preterm birth. These data argue that further examination of β3 adrenergic receptor signaling in myometrium may reveal mirabegron as a useful tocolytic in combination tocolysis regimens.

## Introduction

Approximately 15 million preterm births (PTB) before 37 completed weeks of gestation occur annually worldwide [6]. The regulation of birth timing is unknown [43]. The earliest preterm infants are at risk for major disability [40, 48]. In 2018, 10–14% of births in the USA were premature depending on maternal ethnicity [31]. African-Americans are disproportionally affected [32, 39], a recognized health disparity [10, 30], worsened by the CoV-2 pandemic [50]. Indeed, although many cases of PTB are unrelated to any known pathology of mother or fetus, recent examination of women who contract COVID-19 while pregnant face a higher risk of delivering very early [23]. For those pregnant women who have underlying health conditions such as hypertension, diabetes and/or obesity as well as COVID-19 infection, the risk of preterm birth rises 160 percent. The continuing emergence of CoV-2 variants underscores the urgency of developing effective tocolytics.

PTB is a major medical issue. Complications for preterm infants such as blindness, respiratory distress syndrome, jaundice, infections, and brain hemorrhaging [48] are inversely correlated with gestational length. Long-term issues may arise as well, such as chronic lung disease and neurodevelopmental disabilities [29]. There are several risk factors linked to PTL, including: infections such as chorioamnionitis, smoking or the use of illicit drugs, advanced maternal age (> 35 years old), ethnicity of the mother, polyhydramnios, and chronic maternal conditions, such as high blood pressure or diabetes [38]. Spontaneous PTL (sPTL) accounts for half of PTL cases without known cause [12]. Treatments for PTL are ineffective [9, 17, 34, 41] no matter the underlying cause. Developing new tocolytic strategies requires that we understand the myometrial dysfunction(s) that result in preterm labor leading to preterm birth.

The importance of β3 adrenergic receptor (β3AR) in the production of quiescent mediators [13], combined with its upregulation in the myometrium during pregnancy, makes it a prime target for tocolytic strategies [44]. The β3AR was first identified in 1989 [15] and then universally accepted as a subtype of the beta adrenergic receptor family. The β3AR is present in the small intestine, adipose tissue, vascular endothelium, and the smooth muscle of the colon and bladder [11]. The β3AR has also been described in the myometrium and shown to mediate relaxation [44], where it was determined that the β3AR agonist reduced myometrial contractions in monkeys and confirmed the failure of β2AR stimulation by salbutamol to do so. The β3AR subtype differs from β1 and β2 receptors in the third intra-cytoplasmic loop and C-terminal tail, where the β3AR lacks the consensus sequence for phosphorylation by βAR kinase (βARK). βARK mediates β-arrestin recruitment, followed by internalization and homologous β2AR desensitization. Without a consensus sequence for βARK, the β3AR is not readily downregulated [46]. As such, the β3AR response has longer duration of action and is thus better suited as a target for tocolytic strategy [45]. While these data are encouraging, they do not explain the mechanisms underlying β3AR-stimulated relaxation needed to explore novel tocolytic development.

Multiple underlying downstream mechanisms in the myometrium have been associated with the β3AR, including either or both G_i_ and G_s_ pathways, each with unique downstream effects [19, 22]. Commonly, the G_s_ pathway is thought to mediate relaxation in smooth muscle via cyclic AMP accumulation and protein kinase A activation; however, in the case of the myometrium it may not [27]. Previous work from Croci et al*.* imagined the therapeutic potential of β3AR stimulation, but did not explore signaling beyond assumptions that cAMP subserved relaxation [8]. One pathway that has been described is the β3AR antioxidant effects in macrophages of the myometrium [20]. Another pathway involves coupling of the β3AR to BK_Ca_, large conductance calcium-activated potassium channel [14]. BK_Ca_ is the most prominent potassium channel in the myometrium, thought to play a major role in regulating cell membrane potential and myometrial quiescence [1]. The mechanisms underlying β3AR-mediated relaxation in the myometrium are incompletely understood and may be a result of a combination of different pathways involving other targets, such as nitric oxide (NO) generation.

β3AR has been associated with the activation of NO synthase, which in turn generates NO, resulting in vascular smooth muscle relaxation [13]. L-Arginine is transported into endothelial cells where it interacts with endothelial NOS (eNOS) to produce NO [36]. NO activates soluble guanylyl cyclase in the underlying muscle to produce cyclic GMP, which then activates protein kinase G, that phosphorylates proteins including myosin phosphatase to relax vascular smooth muscle [18]. In the myometrium, NO has been shown to relax the tissue in a cGMP-independent manner [3, 4, 7, 25, 26]. Regardless of the NO-mediated relaxation pathway, the role of eNOS in the β3AR response in myometrium will be addressed here because it may be that existing drugs such as the β3AR agonist mirabegron (Myrbetriq™) could be proposed as part of a combination tocolytic.

Biochemical pathways exploited for tocolysis are inadequate [47]. The unique characterization of the β3AR and its relaxation of myometrium render it a viable target for novel tocolytic strategies. The mechanisms behind this effect are yet to be fully comprehended. This study will investigate the potential of mirabegron, a β3AR selective agonist, as a potential tocolytic, and determine the cellular mechanisms underlying β3AR-mediated relaxation.

## Materials and Methods

*Tissue collection*: Pregnant human myometrium tissue collection from singleton pregnancies was carried out under informed consent. We adhered to an inclusion/exclusion and stratification paradigm to allow our results to be harmonized with those of other labs [33]. Within 20 min of removal, tissues are transported by us to the laboratory in cold Krebs buffer containing, in mM, NaCl (118), KCl (4.75), CaCl_2_ (2.5), KH_2_PO_4_ (1.2), NaHCO_3_ (25), MgCl_2_ (1.2), and dextrose (20), and are adjusted to pH 7.4. Tissues were dissected microscopically in thin strips (0.8 l × 0.3w × 0.2d cm), devoid of obvious blood vessels, and used for primary cell cultures, contractile studies, or a portion stored at -150 °C. We collected non-Hispanic white, Hispanic, and Native American (Table 1). We collect all de-identified data on the treatments and pregnancy course prior to surgery as well as the presence and degree, or absence of infection, mother’s BMI, sex of the infant, smoking status of the mother, and social indicators including socioeconomic status, prenatal care, and maternal education when available. Patient identifiers are not collected; case numbers are used for reference. Exclusion criteria include age < 18 years, any history of drug abuse, long-term use of tocolytics > 48 h (to control confounding factors), comorbid diagnoses such as chorioamnionitis, HIV infection or AIDS, hepatitis C infection, rupture of membrane (PROM/PPROM), uncontrolled diabetes, renal disease, preeclampsia, intrauterine growth restriction (IUGR), and any use of steroids other than dexamethasone/ betamethasone.

**Table 1:** Demographic information for patient samples

Myometrial Tissue State	Age (Years)	White	Hispanic	Native American	Diagnosis
Hysterectomy	26–48	12	2	0	Menorrhagia (6), Dysmenorrhea (3), Uterine Fibroids (2), Abnormal Uterine Bleeding (1), Omental Mass (1), Endometriosis (1)
Term Pregnant (37–39 weeks)	20–40	20	3	0	Repeat C/S (15), Breech (5), Fetal Macrosomia (2), Low Fetal HR (1)
Term in Labor (37–39 weeks)	22–42	13	1	0	Repeat C/S (5), Fetal Distress (3), Failure to Descend (2), Low Fetal HR (1), Uterine Fibroids (1), Fetal Macrosomia (1), Thin Uterine Lining (1)
Preterm (28–34 weeks)	21–37	4	1	1	Preeclampsia (2), Placenta Previa (2), PROM (1), CIN (1)
Preterm in Labor (27–36 weeks)	20–40	3	3	0	Breech (2), Placenta Previa (1), Repeat C/S (1), Placenta Accreta (1), Fetal Position (1)

*Contractile studies:* Dissected strips of myometrium are hung in three 4-channel horizontal tissue bath systems (Danish Myo Technology 820MS, Hinnerup, Denmark), while they are submerged in oxygenated (95% O_2_ and 5% CO_2_) Krebs buffer at 37 °C. The strips are attached to transducers in each bath and pulled to 2 gms tension. KCl (60 mM) was applied for 3 min to stimulate contractions, followed by a washout and a one-hour equilibration period. After spontaneous contractions were established, myometrial strips were treated with 8 nM oxytocin to achieve maximal contractions. Transducer voltages were converted to digital signals and transferred to the LabChart software (ADInstruments) for analysis [3]. Tissues were treated with mirabegron (MBG) (Tocris, Bristol, UK) for one-hour, or with a combination of Nω-nitro-L-arginine (L-NNA) (Sigma-Aldrich, St. Louis, USA), SR59230A (Tocris, Bristol, UK), or paxilline (Sigma-Aldrich, St. Louis, USA) 15 min prior to MBG. Larger doses of MBG were not used to avoid a 1% concentration of DMSO in organ baths. Washouts were performed after each dosing period to observe a return in contractions. Tissues that did not respond to the KCl-challenge or did not display any post-washout contractions at the end were not included.

*Western blot:* Tissue samples were crushed using a mortar and pestle precooled in LN_2_. Crushed tissue samples were added to MAPK buffer containing in mM, (Tris–HCL pH 6.8 (60), glycerol (1%), SDS (2%), leupeptin (0.001), EGTA (1) EDTA (1), AEBSF (1), Na_3_VO_4_ (1), and protease/phosphatase inhibitors (Sigma-Aldrich, St. Louis, USA) followed by 5 min in a tissue homogenizer on ice. For cell protein lysates, cells are trypsinization for 5 min, centrifuged for 10 min (650 RCF) and the cell pellet resuspended in MAPK buffer with sonication on ice for 3 min. Protein quantification of both tissue and cell protein lysates were performed via EZQ (Thermo Fisher, Waltham, USA). For both the β3AR and eNOS protein lysate, 40 μg of protein was loaded into a 4–15% Mini-Protean TGX precast gel in standard tris/glycine PAGE buffers (Bio-Rad, Hercules, USA), and proteins separated at 200 V, and transferred to nitrocellulose blocked in Licor blocking buffer for 1 h. Blots were labeled with either primary mouse anti-β3AR (1:1000, sc-515763, Santa Cruz Biotechnology, Dallas, USA) or primary mouse anti-eNOS (1:1000, ab76198, Abcam, Cambridge, UK), and then labeled with secondary Alexa Fluor 680 donkey anti-mouse (1:25,000, ab175774, Abcam, Cambridge, UK). Both beta-3 adrenergic receptor and eNOS were normalized to their respective GAPDH concentrations in each sample using the primary rabbit anti-GAPDH (1:1000, 2118S, Cell Signaling Technology, MA, USA) and the secondary IRDye 800 donkey anti-rabbit (1:25,000, 926–32,213, Licor Biotechnology, Lincoln, USA).

*Cell culture:* Enzyme solution was prepared containing 20 mg collagenase, 10 mg Trypsin (27,250–018, Gibco, Waltham, USA), and 10 ml of solution containing 5 ml of MACS buffer and 5 ml of Gibco Dulbecco’s Modified Eagle Medium (DMEM, 11,995–065, Gibco, Waltham, USA). The resulting solution was incubated at 37 °C with small, dissected pieces of myometrium in three successive agitation steps (90 s) followed by two 45-min incubation periods at 37 °C with rotation. The digestion is triturated × 3 and filtered through a 100-micron sterile mesh. Cells derived in this fashion are grown to 80% confluency and then preincubated with FcR blocking reagent, then separated over CD31 + bead LS columns using a MidiMACS separator (Miltenyi BioTec, Bergisch Gladbach, Germany) into CD31 + pregnant human myometrial endothelial cells (phMEC) and CD31-pregnant human uterine smooth muscle cells (phUSMC). Once 80% confluent in P0 culture, cell culture is grown to P3 and employed or frozen for future experiments at 1 × 10^6^ cell/ml. Telomerized human uterine smooth muscle cells (hTRT) [21] were grown between P20-P30. The phMEC were grown in endothelial basal medium 2 (PromoCell, St. Louis, USA) containing 10% FBS and 1% penicillin, while the phUSMC and hTRT cells were grown in Gibco DMEM (Gibco, Waltham, USA) containing 10% FBS and 1% penicillin.

*Immunofluorescence:* Cells from CD31 + bead separation were plated on 35-mm glass-bottomed dishes (MatTek Corporation, Ashland, USA) and grown to 70% confluence. Term nonlaboring dissected tissue was cryosectioned at 15 microns onto a 1″ × 3″ × 1.00 mm Snowcoat X-tra microslide (3,800,210, Leica Biosystems, Wetzlar, Germany). Tissue and cells are incubated for 15 min with 4% paraformaldehyde, followed by 0.5% triton-x for 5 min and 5% BSA blocking buffer for 1 h with three PBS washes between steps. Cells and tissue were then stained with an anti-β3AR antibody (1:100, sc-515763, Santa Cruz Biotechnology, Dallas, USA) and anti-CD31 antibody (ab949, Abcam, Cambridge, UK). Following primary staining, cells were stained with secondary antibody (Alexa Fluor 594 ab150080, Abcam, Cambridge, UK). Tissue was stained with secondary antibody for anti-CD31 antibody (Alexa Fluor 594 ab150080, Abcam, Cambridge, UK) and anti-β3AR antibody (Alexa Fluor 488 ab150113, Abcam, Cambridge, UK). Both tissue and cells were imaged under confocal microscopy (10X magnification). In addition to the primary proteins of interest, cells were also stained with wheat germ agglutinin (WGA) conjugated to Alexa Fluor 488 (W11261, Thermo Fisher Scientific, Waltham, USA) to establish cell boundaries, followed by DAPI mounting medium (H-1500, Vector Laboratories) to identify the nucleus. All images were taken with negative controls (lack of experimental condition primary antibody) to verify the absence of nonselective binding (data not shown).

*DAF-FM diacetate assay:* Selected phMEC and phUSMC, along with hTRT cells, were passed onto a half area, 96-well microplate (675,076, Greiner Bio One, Kremsmünster, Austria) at 4,000 cells/well. Cells are grown to 80% confluence. The phMEC were washed x-three with phenol red free EGM-2 media (C-22216, PromoCell, St. Louis, USA), while the phUSMC and hTRT cells were washed with phenol red free DMEM (21,063,029, Thermo Fisher, Waltham, USA). Cells were then incubated with DAF-FM (100 μM) (Ex/Em 495/515, D23844, Thermo Fisher, Waltham, USA) for 30 min at 37 °C and washed, and fresh phenol red free medium was added and incubated for an additional 30 min at 37 °C to allow for complete de-esterfication of intracellular diacetates. phUSMC and phMEC were treated with MBG (100 μM) or left alone as control cells and then processed on the VICTOR Nivo Multimode Microplate Reader (PerkinElmer, Waltham, USA) for 1 h at 37 °C. Only wells with live cells within 2 standard deviations from the mean for each condition were used for statistical analysis. hTRT cells were treated with S-nitrosoglutathione (300 μM) (GSNO, A.G. Scientific, San Diego, USA) as positive controls to verify the DAF-FM diacetate assay (data not shown).

*Statistical analysis:* Experiments involving contractile studies employed 3–5 patients for each data set. Replicate variation was controlled by multiple tissue strips from each patient. Contractile data are presented as area under the curve (AUC) by calculating the integral relative to the minimum baseline of the respective contraction. The last three contractions in the last 15 min for each dosing period was analyzed. Each tissue strip was double normalized, first to itself prior to dosing, and then to a control tissue strip at the respective data collection time point. Control tissue strips were dosed with volume equivalents of the drug (DMSO for MBG, SR59230A, paxilline). The average of the AUC values from the control tissue strips was set to 100% so that data from experimental strips would show percentage relative to baseline. In the western blot experiments, each sample was from a different patient, representing its own ‘n.’ Statistical analysis for contractile studies and western blots were completed using two-tailed, unpaired t-tests. In the DAF-FM diacetate assay, each individual well containing cells was considered its own ‘n.’ Both experimental and control conditions for the cells had results from a baseline of wells filled with DAF-FM and media alone subtracted from the conditions. Afterward, the untreated conditions were subtracted from the treated conditions for each cell type. Then a 0 to 1 normalization was performed for the average of the phUSMC, and the cells were normalized against it to measure relative fold change in fluorescence. Each individual time point was compared vertically to its corresponding time point in their cell type, and a two-tailed, unpaired t-test was performed to detect significance. Data points within each cell type were compared to each other using a one-way ANOVA to detect significance within their respective group. All statistical analysis was done using Prism (version 8.4.3, GraphPad Software, San Diego, USA) with significance set to p < 0.05.

## Results

### β3AR and eNOS Expression in Disparate States of Pregnancy

**Fig. 1:**
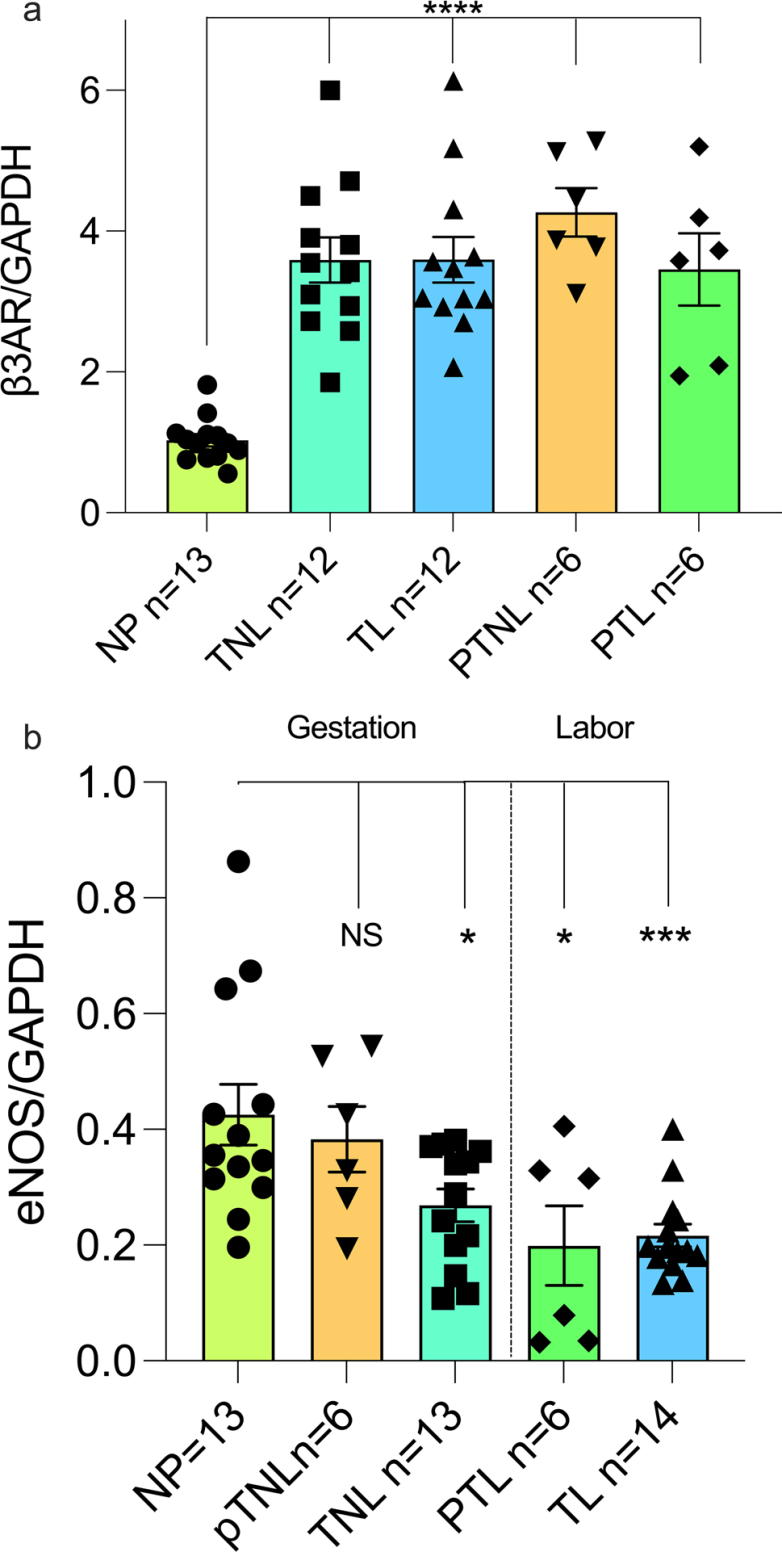
β3AR and eNOS expression in disparate states of pregnancy A.) The β3AR increases throughout pregnancy in PTNL (*p* < 0.0001), PTL (*p* < 0.0001), TNL (*p* < 0.0001), and TL (p < 0.0001) compared to a NP state. There is no difference between the different states of pregnancy (*p* > 0.05). B.) eNOS expression decreases as gestation time increases (PTNL *p* > 0.05, TNL *P* < 0.05), and decreases in the state of labor regardless of gestation time (PTL *P* < 0.05, TL *p* < 0.001) compared to a NP state

Gene expression has been shown to vary widely in the myometrium of women who experience preterm labor, thus amplifying the need for understanding the expression of target proteins, such as β3AR and eNOS, to determine whether their expression indicates a vital role in mediating labor [3, 16, 24, 37]. β2AR expression has been shown to decrease in the human myometrium after stimulation with beta mimetics [5]; however, the β3AR does not desensitize like the β2AR, and the β3AR is the dominant beta adrenergic receptor in the human myometrium [44, 45]. We examined myometrial β3AR expression in disparate states of pregnancy for the first time. Western blot was performed using protein lysates from human myometrial tissue samples nonpregnant (NP) (*n* = 13), term nonlabor (TNL) (*n* = 12), term labor (TL) (*n* = 12), preterm nonlabor (PTNL) (*n* = 6), and PTL (*n* = 6), and each sample was normalized to their respective GAPDH concentration (Fig. 1a). Results showed an increase in β3AR concentration during pregnancy compared to the nonpregnant state (*p* < 0.0001). During gestation and labor there was no difference in β3AR expression (p > 0.05). This increase in β3AR expression occurring as a result of pregnancy suggests its importance as a target for promoting quiescence during gestation.

To investigate the role of β3ARs in NO production, western blot was performed to determine the relative amount of eNOS expressed in human myometrial tissue samples from NP (*n* = 13), TNL (*n* = 13), TL (*n* = 14), PTNL (*n* = 6), and PTL tissue samples (*n* = 6) and each sample was normalized to GAPDH (Fig. 1b). Results showed that there was no observed difference in eNOS expression in PTNL compared to NP samples (*p* > 0.05), but decreased as gestation time increased to term (*p* < 0.05). Also, there is a decrease in concentration of eNOS in term laboring (*p* < 0.001) and preterm laboring (*p* < 0.05) samples compared to NP samples.

## Effect of Mirabegron on Human Myometrial Tissue

**Fig. 2:**
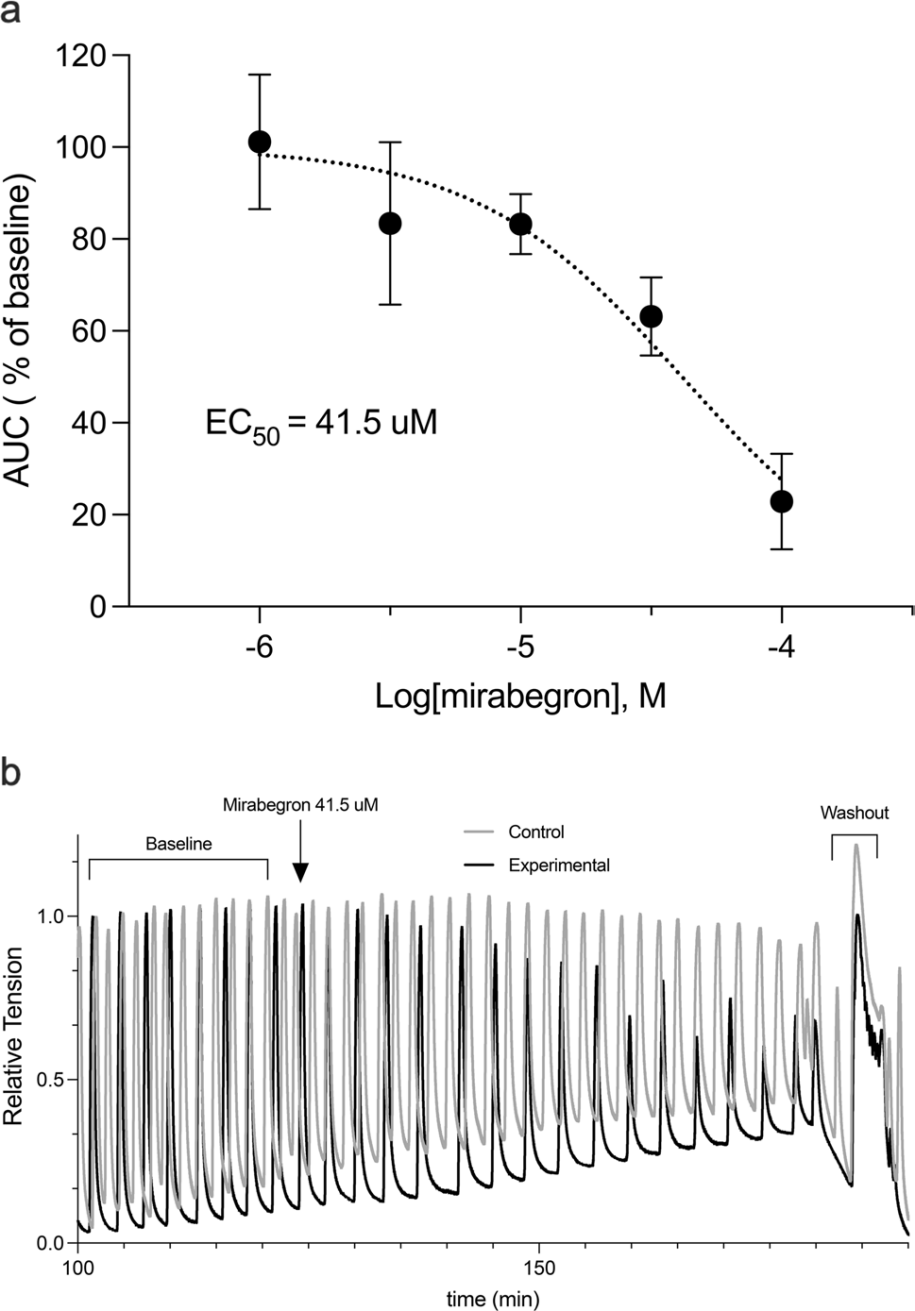
EC_50_ of mirabegron on Human Myometrial Tissue **A**.) Human myometrial tissues (TNL) were challenged with KCl and given oxytocin (8 nM) to maximize baseline contractions. Tissues were dosed with mirabegron for 1 h at half-log doses ranging from 1 μM to 100 μM (*n* = 5). The AUC relative to the percentage of the baseline for each dose was plotted, showing a EC_50_ of 41.5 μM. **B**.) Depicts a trace of human myometrial tissues (TNL) being dosed with mirabegron (41.5 μM) or volume equivalent DMSO (control) relative to the maximum contractions established in the baseline using oxytocin. Trace depicts an effective EC_50_ along with a return in contractions during the last washout

MBG is an FDA-approved β3AR selective agonist used to treat overactive bladder syndrome. MBG has been shown to relax smooth muscle on contracting bladder strips [49]. Thus, we examined the effects of MBG on human myometrial tissue strips in TNL patients. Dose response was performed to determine the EC_50_ for MBG in human myometrium (Fig. 2a). After the myometrial tissue strips reached maximum contractile activity following 8 nM oxytocin, they were treated with MBG in half-log doses ranging from 1 μM to 100 μM, or a volume equivalent of MBG diluent, DMSO (*n* = 5). Analysis using the AUC resulted in a MBG-mediated EC_50_ of 41.5 μM. Traces of contracting uterine smooth muscle treated with 41.5 μM MBG and its volume equivalent buffer control, each compared to their respective baseline, showed a drop in about 50% AUC and a return of contractions following washout (Fig. 2b).

**Fig. 3:**
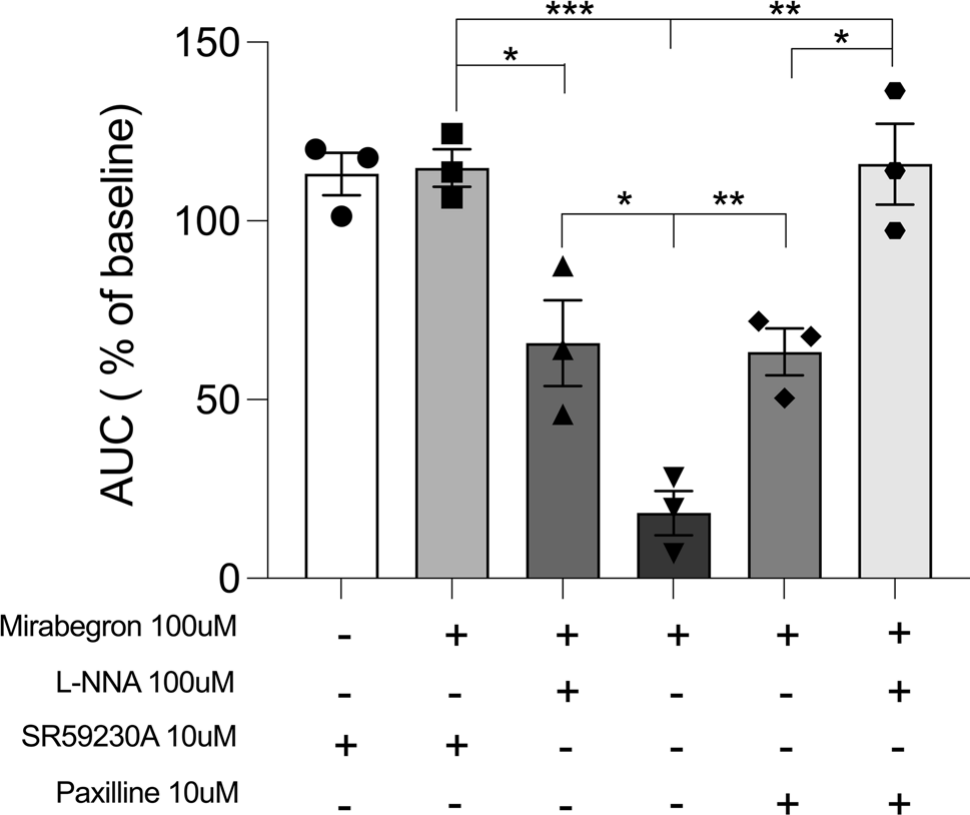
Inhibition of mirabegron through different mechanistic pathways – AUC presented as relative to the percentage of baseline. SR59230A ± mirabegron show no difference in AUC (*p* > 0.05). Mirabegron had a significantly lower AUC than SR59230A + mirabegron (*p* < 0.001), L-NNA + mirabegron (*p* < 0.05), paxilline + mirabegron (*p* < 0.01), and mirabegron in combination with L-NNA + paxilline (*p* < 0.01). There was a distinction in AUC between mirabegron + SR59230A and mirabegron + L-NNA (*p* < 0.05). Also, there was a distinction between mirabegron + paxilline and mirabegron in combination with L-NNA + paxilline (*p* < 0.05). Mirabegron + SR59230A showed no difference in AUC compared to mirabegron in combination with L-NNA + paxilline (*p* > 0.05)

To determine the mechanistic pathways responsible for the observed relaxation of oxytocin contractions, TNL tissue strips were pretreated with either 10 μM SR59230A (a selective β3AR antagonist, *n* = 3), 100 μM L-NNA (NOS inhibitor, *n* = 3), 10 μM paxilline (BK_Ca_ inhibitor, *n* = 3), a combination of 100 μM L-NNA and 10 μM paxilline (*n* = 3), or untreated (8 nM oxytocin alone) for 15 min. Following this pretreatment period, tissues were dosed with 100 μM MBG, except for tissues pretreated with SR59230A, which were either given 100 μM MBG or left untreated. Control organ baths were dosed with volumetric equivalent DMSO. Results were graphed against the percentage of AUC relative to the baseline for their respective conditions (Fig. 3). Tissues treated with SR59230A alone showed 113% in AUC relative to baseline, SR59230A plus 100 μM MBG showed a 115% AUC relative to baseline. MBG (100 μM) exhibited an AUC of 18.27% relative to baseline, and 100 μM MBG plus L-NNA demonstrated an AUC of 65.83% relative to baseline. Furthermore, 100 μM MBG plus paxilline showed an AUC of 63.3% relative to baseline. A combination of MBG, L-NNA, and paxilline had an AUC of 116% relative to baseline. There was no difference when comparing the AUC of SR59230A and SR59230A with MBG (*p* > 0.05). Tissue treated with SR59230A plus MBG showed a difference in AUC compared to the combination of MBG and L-NNA (*p* < 0.05) and showed a difference in AUC to MBG alone (*P* < 0.001). Tissue dosed with a combination of L-NNA and MBG showed a difference in AUC to tissue dosed with only MBG (*p* < 0.05). There was a distinction between the AUC in tissue treated with MBG alone and MBG combined with paxilline (*p* < 0.01). When tissue strips were coadministered MBG, L-NNA, and paxilline, there was a clear contrast in AUC compared to MBG alone (*p* < 0.01). There was still a difference in AUC between MBG plus paxilline and MBG combined with paxilline and L-NNA (*p* < 0.05). Lastly, there was no significant change in AUC when comparing MBG plus SR59230A and MBG plus paxilline and L-NNA (*p* > 0.05).

## β3AR Expression in phMEC and phUSMC

**Fig. 4:**
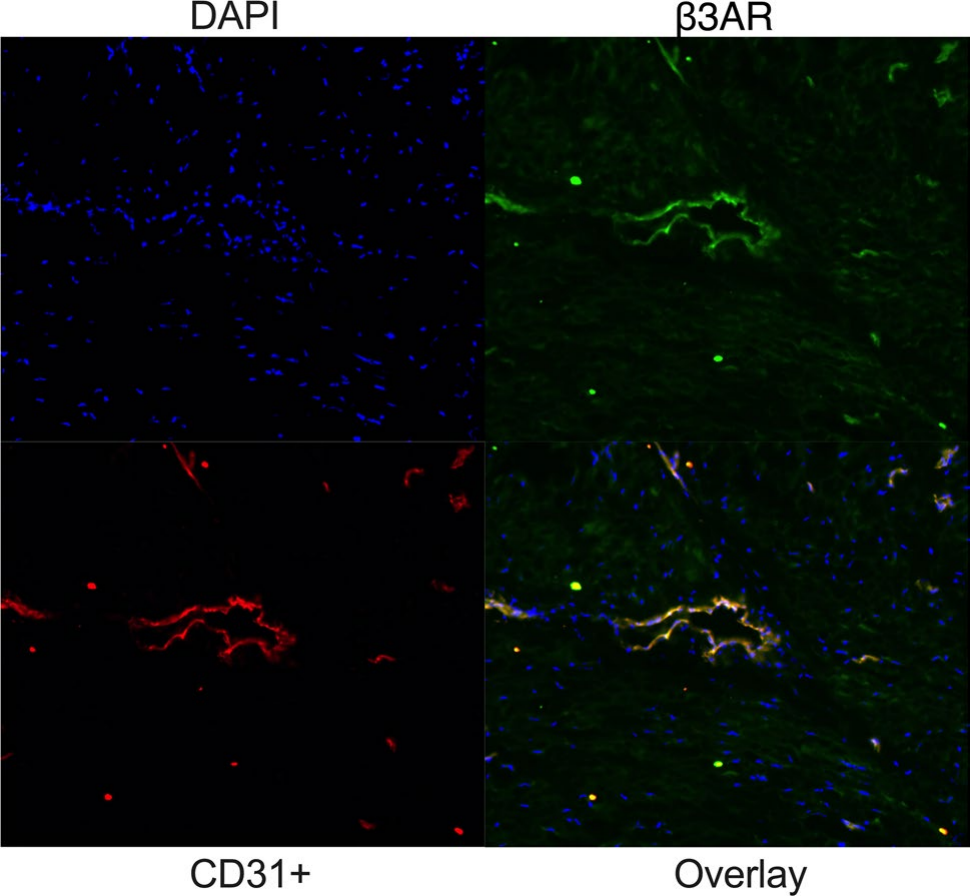
Detection of β3AR and CD31 + in TNL tissue – TNL myometrial tissue was cryosectioned at 15 microns and stained with DAPI, β3AR, and CD31 + antibodies. Immunofluorescence confirms that both β3AR and CD31 + cells are present in the tissue. β3AR is colocalized with CD31 + cells, but is also expressed in the surrounding tissue as well

**Fig. 5:**
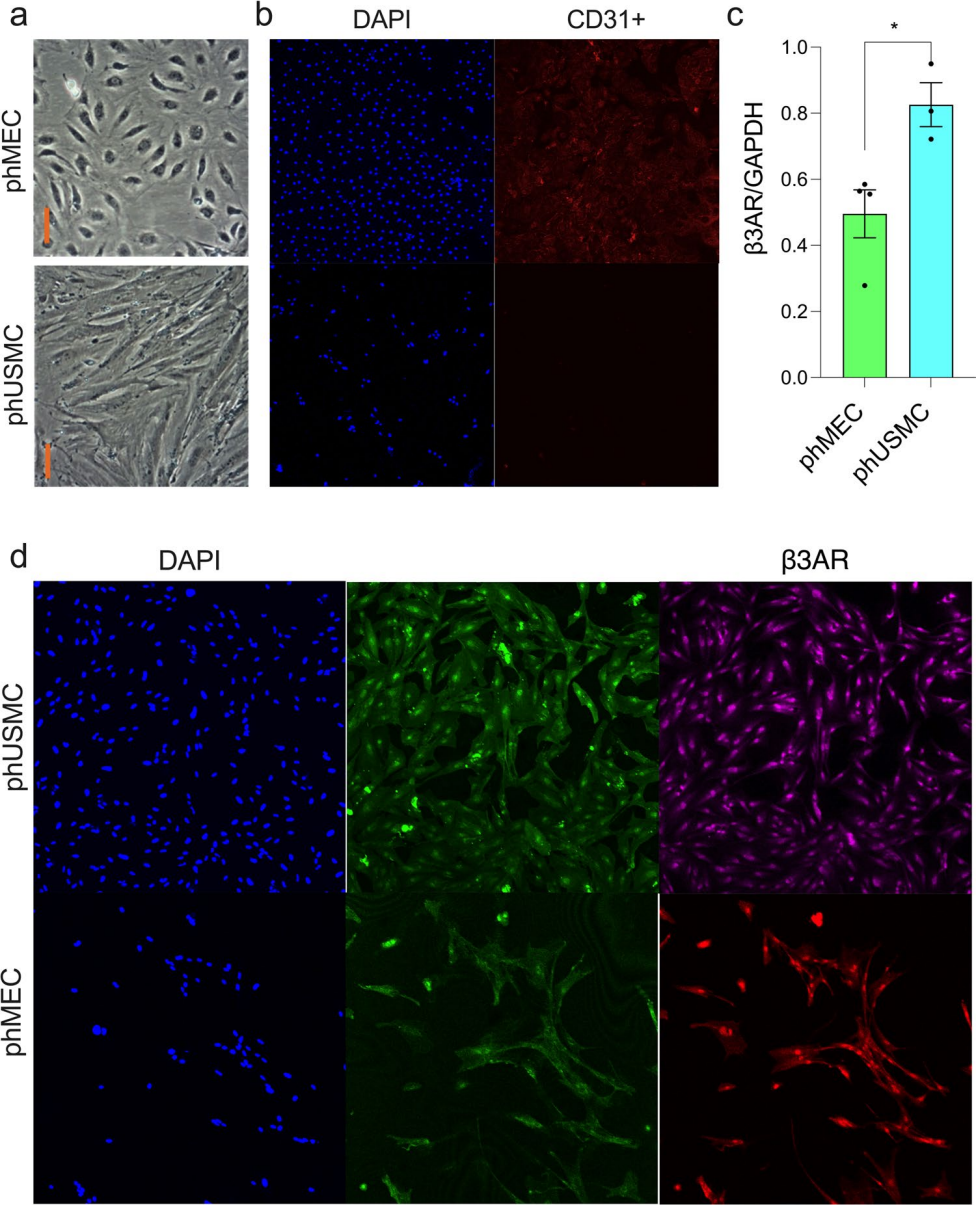
Detection and quantification of β3AR in phMEC and phUSMC—Primary cells of the myometrium (TNL) were grown out in tissue culture and selected into phMEC and phUSMC (P3). **A**.) Micrograph images of phMEC (Top) and phUSMC (bottom) from TNL primary cells on day 3 following selection. Orange bar insert = 100 micron. **B**.) Both phMEC and phUSMC were stained with DAPI and primary CD31 + antibodies with secondary Alexa Fluor 594 antibodies. phMEC expresses CD31 + cells, while phUSMC shows no expression of CD31 + cells. **C**.) Western blot confirms quantifiably the expression of β3AR in both phMEC (*n* = 4) and phUSMC (*n* = 3), and depicts a larger β3AR expression in phUSMC versus phMEC (*p* < 0.05). **D**.) phMEC and phUSMC were stained with DAPI, WGA conjugated with Alexa Fluor 488 (cellular membrane), and primary β3AR antibodies with secondary Alexa Fluor 594 antibodies. The WGA images display the phenotype expected from phMEC and phUSMC, and both phMEC and phUSMC express the β3AR

Immunofluorescence was performed using tissue (TNL) and primary myometrial cells (TNL, P3) following CD31 + selection to isolate phMECs from phUSMCs. Cryosectioned tissue was stained with both CD31 + and β3AR antibodies. Imaging revealed colocalization of β3AR and CD31 + , and high levels of β3AR expression in surrounding tissue (Fig. 4). Micrograph images were taken showing isolated endothelial (top) and myocyte (bottom) cells from TNL primary culture (Fig. 5a). Immunofluorescence was performed to confirm the selection of phMEC versus phUSMC. Cells were stained with DAPI and CD31 + primary and Alexa Fluor 594 secondary antibodies. Images depicted that CD31 + cells were identified in phMEC and not phUSMC (Fig. 5b). Both phMEC and phUSMC were labeled with DAPI to detect the nucleus, wheat germ agglutinin (WGA) conjugated to Alexa Fluor 488 to visualize the cell membrane, and with primary β3AR and secondary Alexa Fluor 594 antibodies (Fig. 5d). Bright-field microscopy demonstrated the expected cellular phenotype for the phMEC and phUSMC in the WGA stain, and it depicted β3AR in both the phMEC and phUSMC compartments.

After obtaining images of the β3AR in phMEC and phUSMC, we next quantified the expression of the receptor in each cellular compartment. Western blot was performed in cellular lysates of phMEC (*n* = 3) and phUSMC (*n* = 3), with each sample being normalized to their respective GAPDH concentration (Fig. 5c). Results showed a larger β3AR concentration in phUSMC compared to phMEC (*p* < 0.05).

## Nitric Oxide Production in phMEC versus phUSMC through the β3AR

It has been well established that the β3AR is associated with NO production in vascular smooth muscle [13] and that NO can relax the myometrium [3, 25, 26]. Thus, we next aimed to determine whether the stimulation of the β3AR can increase NO production in the myometrium, and if so, in which cellular compartment.

**Fig. 6:**
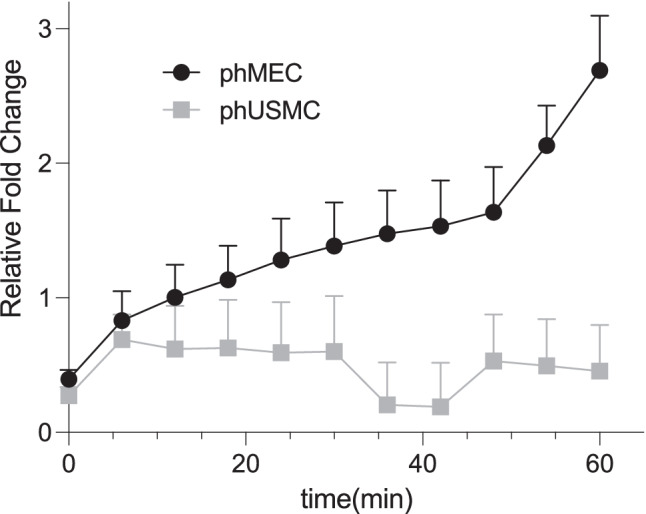
DAF-FM detection in mirabegron treated phMEC and phUSMC – phMEC (*n* = 12) and phUSMC (*n* = 18) were preincubated with DAF-FM (30 min) then dosed with 100 µM mirabegron ( ±). NO generation (DAF-FM fluorescence 495/530) was measured in 6-min intervals for 1 h. Only wells with live cells within 2 standard deviations from the mean for each condition were used for statistical analysis. Data is plotted as a relative fold increase in fluorescence over untreated control. NO generation in phMEC cells increased significantly over phUSMC at all time points ≥ t = 36 min (*p* < 0.05). One-way ANOVA confirms no difference between the phUSMC data points but a significant difference between the phMEC data points (*p* < 0.0001)

In order to quantify the amount of NO being produced from β3AR, cells were preincubated with DAF-FM diacetate, which has been shown to detect intracellular NO [28]. NO production was quantified in phMEC and phUSMC following β3AR stimulation. All cells were incubated with DAF-FM for 30 min, followed by a 30 min post-media change incubation to allow for de-esterfication. Both phMEC and phUSMC were each split into two groups, one group was administered MBG (100 μM), while the other was left untreated. Data was recorded at 11 time points ranging from t = 0 min to t = 60 min in 6-min intervals. The phUSMC (*n* = 18) and phMEC (*n* = 12) were plotted as a relative fold increase over the untreated control and normalized against the average of the phUSMC (Fig. 6). No significant increase in NO generation was seen in phUSMC, while a significant increase in NO production was seen in phMEC data (*p* < 0.0001). There was a significant increase in NO production in the phMEC over the phUSMC at all time points ≥ t = 36 min (*p* < 0.05).

## Discussion

Preterm labor continues to be the leading cause of morbidity and mortality in neonates [35, 42, 48]. Canonical pathways employed by current tocolytic strategies have proved to be ineffective [47] at delaying parturition, and it is imperative that novel approaches to tocolytics be developed in order to prevent preterm birth.

Previous studies have shown that the unique characteristics of the β3AR, which include being resistant to the βAR kinase/arrestin downregulation pathway and being abundant in term myometrium [44, 45], make it a potential target for tocolytic approaches. We show that β3AR expression increased in disparate states of pregnancy (Fig. 1). This increase in the β3AR supports the importance of the role it may play in maintaining quiescence throughout gestation and its potential as a target for tocolytic strategy.

The potential of the β3AR as a tocolytic target led us investigate the efficacy of an FDA-approved selective agonist, MBG. MBG is able to mediate relaxation reversibly in contracting human myometrial tissue strips (Fig. 2). Although other β3AR agonists have been examined [2, 45], the effects of MBG shown here are unique. MBG is a Pregnancy Category C drug that could be employed during pregnancy, with no studies being performed that have shown any potential harm to the fetus. MBG is known to have side effects involving hypertension [11], so it would be advised to use cautiously with women that are prehypertensive or preeclamptic. Since MBG is dosed at higher concentrations to mediate contractions (Fig. 2), it may be wiser to incorporate it into tocolytic strategies at lower doses in combination with other therapeutic regimens. More importantly, the effects of MBG support the stimulation of β3AR in developing novel tocolytic strategies.

In order to incorporate MBG and further establish β3AR as a potential tocolytic, we sought to confirm its selectivity and signaling in myometrium. The relaxation effects of MBG (Fig. 3) were completely inhibited by the β3AR inhibitor, SR59230A, which confirms its selectivity for the receptor and partial inhibition of MBG induced relaxation was seen in the presence of either L-NNA or paxilline. Since the β3AR has been shown to be linked to the activation of eNOS [13], partial inhibition from L-NNA showed that the receptor is linked to eNOS activity in the myometrium as well. Data support the importance of eNOS in the myometrium during pregnancy as it decreases in concentration when at term or in labor (Fig. 1b). Paxilline inhibits the effects of the BK_Ca_, confirming previous study findings on the coupling of this potassium channel with the β3AR in vascular tissue [14]. However, these data show that the inhibition of MBG-mediated relaxation is only partial and that relaxation is due to multiple downstream mechanisms. A combination of both paxilline and L-NNA completely inhibited the effects of MBG, similar to that of SR59230A confirming that MBG stimulates the β3AR in disparate cellular compartments in myometrium involving both eNOS- and BK_Ca_-mediated pathways.

The potential of MBG targeting multiple compartments and the crucial role eNOS may play in managing quiescence lead us to investigate whether NO was being generated from β3AR stimulation in phMEC and phUSMC. Our data (Fig. 4, 5) confirm that microvascular endothelium and myocytes can be isolated and that the β3AR is present in both cell types. β3AR expression is greater in phUSMC versus phMEC (Fig. 5c). Using DAF-FM diacetate as a NO detector, we show that MBG increases NO production over a one-hour time period in phMEC but not in phUSMC (Fig. 6). These data verify that NO generation by the β3AR is specific to the microvascular endothelium.

As novel approaches to development of tocolytic strategies continue to be made, the β3AR becomes more of an appealing target. The β3AR is ubiquitous in the myometrium throughout pregnancy. MBG relaxes the myometrium using multiple downstream mechanisms, involving both eNOS and BK_Ca_. The β3AR was identified in both phUSMC and phMEC, while mediating NO generation only in phMEC.

## Data Availability

All data and materials support this study’s claims and comply with field standards.

## Code Availability

All software applications support this study’s claims and comply with field standards.
